# Cerebellar-Dependent Associative Learning Is Preserved in Duchenne Muscular Dystrophy: A Study Using Delay Eyeblink Conditioning

**DOI:** 10.1371/journal.pone.0126528

**Published:** 2015-05-14

**Authors:** Ulrike Schara, Melanie Busse, Dagmar Timmann, Marcus Gerwig

**Affiliations:** 1 Department of Neuropediatrics, Developmental Neurology and Social Pediatrics,University of Duisburg-Essen, Essen, Germany; 2 Department of Neurology, University of Duisburg-Essen, Essen, Germany; University of Minnesota Medical School, UNITED STATES

## Abstract

**Objective:**

Besides progressive muscle weakness cognitive deficits have been reported in patients with Duchenne muscular dystrophy (DMD). Cerebellar dysfunction has been proposed to explain cognitive deficits at least in part. In animal models of DMD disturbed Purkinje cell function has been shown following loss of dystrophin. Furthermore there is increasing evidence that the lateral cerebellum contributes to cognitive processing. In the present study cerebellar-dependent delay eyeblink conditioning, a form of associative learning, was used to assess cerebellar function in DMD children.

**Methods:**

Delay eyeblink conditioning was examined in eight genetically defined male patients with DMD and in ten age-matched control subjects. Acquisition, timing and extinction of conditioned eyeblink responses (CR) were assessed during a single conditioning session.

**Results:**

Both groups showed a significant increase of CRs during the course of learning (block effect p < 0.001). CR acquisition was not impaired in DMD patients (mean total CR incidence 37.4 ± 17.6%) as compared to control subjects (36.2 ± 17.3%; group effect p = 0.89; group by block effect p = 0.38; ANOVA with repeated measures). In addition, CR timing and extinction was not different from controls.

**Conclusions:**

Delay eyeblink conditioning was preserved in the present DMD patients. Because eyeblink conditioning depends on the integrity of the intermediate cerebellum, this older part of the cerebellum may be relatively preserved in DMD. The present findings agree with animal model data showing that the newer, lateral cerebellum is primarily affected in DMD.

## Introduction

Duchenne muscular dystrophy (DMD) is a monogenetic X-chromosome linked recessive disorder with mutations of the dystrophin gene, resulting in a deficient and lowered dystrophin protein [1]. Dystrophin is important for the structural stability of muscles as well as signal transduction and thus progressive muscle weakness occurs in the course of the disease [2]. In addition to wasting of muscles there is emerging evidence that patients with DMD develop behavioral disorders and a moderate to severe mental retardation [3]. Cognitive deficits and speech disturbances in DMD patients have been described already in early reports [4]. More recent data suggest that patients show impaired problem solving, planning and other disturbed higher cognitive and executive functions [5]. Notably, deficits in attention in verbal tasks, sentence repetition and recall of digits, phonological processing and verbal working memory have been reported [6–9]. Also in animals lack of dystrophin was followed by impaired long term object recognition and spatial memory [10]. For a detailed recent review of neuropsychological and neurobehavioral findings in DMD patients the reader is referred to Snow et al. [11].

Cognitive impairment has been related to a loss of dystrophin in neurons of the cerebral cortex, including the temporo-parietal cortex, and the hippocampus, structures which are involved in the processing of various cognitive functions [12–14]. As revealed by studies in dystrophin deficient mdx-mice the largest deficits of dystrophin have been reported in the cerebellar cortex where it is localized along the somata and dendrites of Purkinje cells [15, 16]. A specific link between cognitive disturbances and a possible cerebellar interference in DMD derives from emerging evidence for a role of the cerebellum in cognitive tasks, in particular of the lateral cerebellum [17, 18]. This is supported by anatomical studies showing reciprocal connections of the Ncl. dentatus to the dorsolateral prefrontal cortex and the posterior parts of the parietal cortex [19, 20]. Furthermore, human cerebellar lesion and functional imaging studies strengthen the hypothesis of a cognitive function of the cerebellum [21, 22]. Hence, some authors attributed cognitive deficits in DMD predominantly to an impaired modulating function of the cerebellum [23, 24].

On the cellular level studies in mice have shown that dystrophin appears to be involved in the inhibitory synaptic function and in the induction and extent of synaptic plasticity of Purkinje cells. Lack of dystrophin appears to influence GABA-ergic function, but is also followed by a decreased long-term depression (LTD) at the parallel fibre-Purkinje cell (PF-PC) synapse as revealed by intracellular recordings from Purkinje cells in dystrophin deficient mdx mice [25, 26]. This form of synaptic plasticity has been shown to be of critical importance in cerebellar-dependent learning, in particular eyeblink conditioning, although newer studies show that other forms of plasticity likely contribute [27–29].

The aim of the present study was to examine cerebellar function in subjects with DMD using classical delay conditioning of the eyeblink reflex. In the past decades this form of non-declarative, cerebellar-dependent associative learning has been established as a robust tool to investigate cerebellar disorders in animals and humans [30–33]. An unconditioned stimulus (US), e.g. a corneal air puff is provided near the eye and elicits a reflexive blink, the unconditioned response (UR) that is a closure of the eyelid. When an initially neutral conditioned stimulus (CS), such as a tone, is repeatedly paired with the US, a learned conditioned response (CR) develops. Normally the CR appears well timed such that the eyelid is lowered when the air puff arrives. Beyond patients with distinct cerebellar disorders eyeblink conditioning has been shown useful to detect cerebellar dysfunction, even if subclinical, in other conditions for example in essential tremor [34], in dyslexia [35, 36], in attention-deficit hyperactivity disorder [37] and in patients with migraine [38].

Common DMD patients were recruited from our neuropediatric outpatient clinic irrespective of their cognitive status. Acquisition, timing as well as extinction of conditioned eyeblink responses were analysed and compared to age-matched controls. Considering the proposed cerebellar involvement in cognitive deficits observed in DMD, delay eyeblink conditioning was expected to be impaired in the DMD patients.

## Subjects and Methods

The study was conducted between May 2011 and February 2013. Nine male patients with genetically defined DMD (mean age 12.1 ± 1.5, age range 10–14 years) and 10 sex- and age-matched healthy control subjects (mean age 11.5 ± 1.3, age range 9–13 years) were included. According to the natural history of the disease all boys with DMD manifested at the age of two to three years. One of the patients did not complete the experimental session, therefore data of 8 patients were analysed. Patients were recruited from the outpatient neuromuscular clinic of the Neuropediatric Department of the University of Duisburg-Essen and control subjects by contacting pupils and their parents of a primary or middle school. The study was approved by the local ethics committee and written informed consent was obtained from the subjects and their legal representatives. To determine muscle weakness and possible cerebellar signs or ataxia symptoms a neurological examination was conducted by an experienced neuropediatrician, U.S. or M.B. None of the control subjects had a history of neurological diseases, they were free from any medication and there were no neurological signs in controls on examination.

In DMD patients muscle strength was examined according to the Medical Research Council Scale (MRC) [39]. Because muscle weakness was present in all patients and influenced performance on limb coordination, balance and gait testing, use of ataxia scales was not meaningful to determine the severity of cerebellar signs. Seven patients were not able to walk without support. None of the patients had to use a respirator or was provided with a percutaneous endoscopic gastrostomy (PEG). Five of the patients were currently treated with corticosteroids (Deflazacort or Prednisone 0.45–0.75mg/kg/d). The dosages were lower than current international standards due to individual clinical conditions like development of obesity or loss of ambulatory ability. No other medication was applied. Further clinical and genetic characteristics of the patients are summarized in Table 1.

**Table 1:** Clinical characteristics and X-chromosomal genetic findings in DMD patients.

*Patient/Clinical characteristics*	1	2	3	4	5	6	7	8
***Age (years)***	12	12	14	10	13	10	13	11
***Mutation***	*Deletion 51*	*Deletion 46–51*	*Deletion 45–48*	*Deletion 45–52*	*Point Mutation Exon 14*	*Point Mutation Exon 51*	*Deletion 10–11*	*Deletion 48–52*
***Respiratory disorder***	+	*no*	++	++	++	*no*	+	++
***Cardiomyopathy***	*no*	*no*	+	*no*	+	*no*	*no*	*no*
***Corticosteroids***	*yes*	*no*	*yes*	*yes*	*yes*	*no*	*no*	*yes*
***Cerebellar signs***								
*Oculomotor Dis*	*no*	*no*	*no*	*no*	*no*	*no*	*no*	*no*
*Nystagmus*	*no*	*no*	*no*	*no*	*no*	*no*	*no*	*no*
*Dysarthria*	*no*	*no*	*no*	*no*	*no*	*no*	*no*	*no*
*Diadochokinesis*	*Brady-*	*Brady-*	*Dys-*	*Brady-*	*Brady-*	*Brady-*	*n*.*a*.	*Brady-*
*Finger to Nose*	*eumetric*	*n*.*a*.	*eumetric*	*n*.*a*.	*eumetric*	*eumetric*	*n*.*a*.	*n*.*a*.
*Heel to Shin*	*unsteady*	*n*.*a*.	*n*.*a*.	*n*.*a*.	*unsteady*	*unsteady*	*n*.*a*.	*n*.*a*.
*Romberg*	*unsteady*	*n*.*a*.	*n*.*a*.	*n*.*a*.	*n*.*a*.	*n*.*a*.	*n*.*a*.	*n*.*a*.
*Gait*	*slow*	*not possible*	*not possible*	*not possible*	*not possible*	*not possible*	*not possible*	*not possible*
***Muscle strength*** *(MRC grade)*								
*Handgrip*	4	4	4	4	4	4	4	4
*Raise shoulder*	4	4	3	3	3	4	2	4
*Abduction Arm*	4	4	3	2	3	4	2	4
*Adduction Arm*	4	4	3	2	3	4	2	4
*Abduction of Leg*	4	2	2	2	2	4	2	3
*Adduction of Leg*	4	2	2	2	2	4	2	3
*A*.*dorsiflexion*	4	3	3	3	3	4	3	4
*A*.*plantarflexion*	4	3	3	3	3	4	3	4
***Contraction***								
	*Ankle*	*Ankle*	*Ankle*	*Ankle*	*Ankle*	*Ankle*	*Ankle*	*Ankle*
		*Elbow*	*Knee*	*Knee*	*Knee*	*Knee*	*Knee*	*Knee*
				*Hip*	*Hip*	*Elbow*	*Hip*	*Hip*
					*Thumb*		*Elbow*	

Examination of limb coordination, balance and gait and the use of ataxia scales to determine the severity of cerebellar signs was not meaningful in case of muscle weakness. Muscle strength was examined according to the graduation of the Medical Research Council (MRC). Abbr.: + = mild, ++ = moderate; A. = Ankle; Dis. = Disorder; n.a. = not applicable in case of muscle weakness. See *Methods*for further details.

At the beginning of the experiment hearing thresholds were determined in each subject using 1 KHz, the frequency of the CS. Thresholds of both ears (dB SPL) were within normal age limits in all participants, there was no significant difference between patients and controls. None of the participants suffered from eye diseases.

### Eyeblink conditioning

As reported previously in detail a standard delay eyeblink conditioning paradigm was used [40, 41]. All subjects were investigated by the same investigator (M.B.) in a quiet room, seated assured and comfortably on a chair and watching a silent movie. At the beginning ten CS alone and ten US alone trials were presented in an unpaired and random order, this was followed by 100 paired CS-US trials and then 10 CS alone extinction trials. The US consisted of an air puff (duration 100ms, intensity 400 KPa at source, 110 KPa at nozzle), directed near the outer canthus of the right eye at a distance of about 10 mm. As the CS a tone (1 KHz; 70 dB sound pressure level, SPL; duration 540ms) was presented ipsilaterally and coterminated with the air puff. Surface EMG recordings were taken from orbicularis oculi muscles bilaterally.

In paired and extinction trials CRs were semiautomatically identified in the CS-US interval using custom made software [42]. The onset of a CR was defined where EMG activity reached 7.5% of the EMG maximum in each recording with a minimum duration of 20ms and a minimum integral of 1 mV*ms. Trials were visually inspected and implausible identification of CRs was manually corrected. Responses occurring within the 150ms interval after CS onset were considered as reflexive responses to the tone (i.e. alpha responses) and not as CRs [43]. Trials with spontaneous blinks occurring prior to CS onset were excluded from the analysis.

As a measure of learning the primary outcome parameter was CR acquisition. The number of CRs was expressed as the percentage of trials containing responses with respect to each block of ten trials (percentage CR incidence) and the total number of trials (total percentage CR incidence).

As secondary outcome measures, timing and extinction of CRs were assessed. As outlined above onset and peaktime of CRs in paired trials and URs in unpaired trials were automatically quantified. US onset was set as 0 ms. CR onset and peaktime were expressed as negative values i.e. prior to onset of the US [41]. EMG amplitudes were not analyzed due to methodological limitations in surface EMG recordings.

As a measure of extinction the CR incidence within the last block of paired trials (block 10) was compared with the extinction block. All subjects exhibited at least one CR during extinction ensuring a sufficient ability of learning in the paired trials [42].

The frequency of spontaneous blinks was measured in each session within one minute both at the beginning and in the end of the experiment. The rate of alpha-blinks was analysed within the 150 ms interval after CS onset of 100 paired trials.

### Data analysis

Analysis of variance with repeated measures (ANOVA) was calculated in paired trials with percentage CR incidence as dependent variable, block (1–10: ten blocks of ten paired trials) as within subject factor and group (controls vs. DMD patients) as between subject factor. Level of significance was set at p < 0.05. For all effects, the degrees of freedom were adjusted, if appropriate, according to Greenhouse-Geisser [44]. In addition, ANOVA was calculated comparing the last block of paired trials and the block of extinction trials. CR and UR timing parameters, spontaneous blink rates and alpha were compared between controls and patients using unpaired *t*-tests.

## Results

### CR acquisition

**Fig 1:**
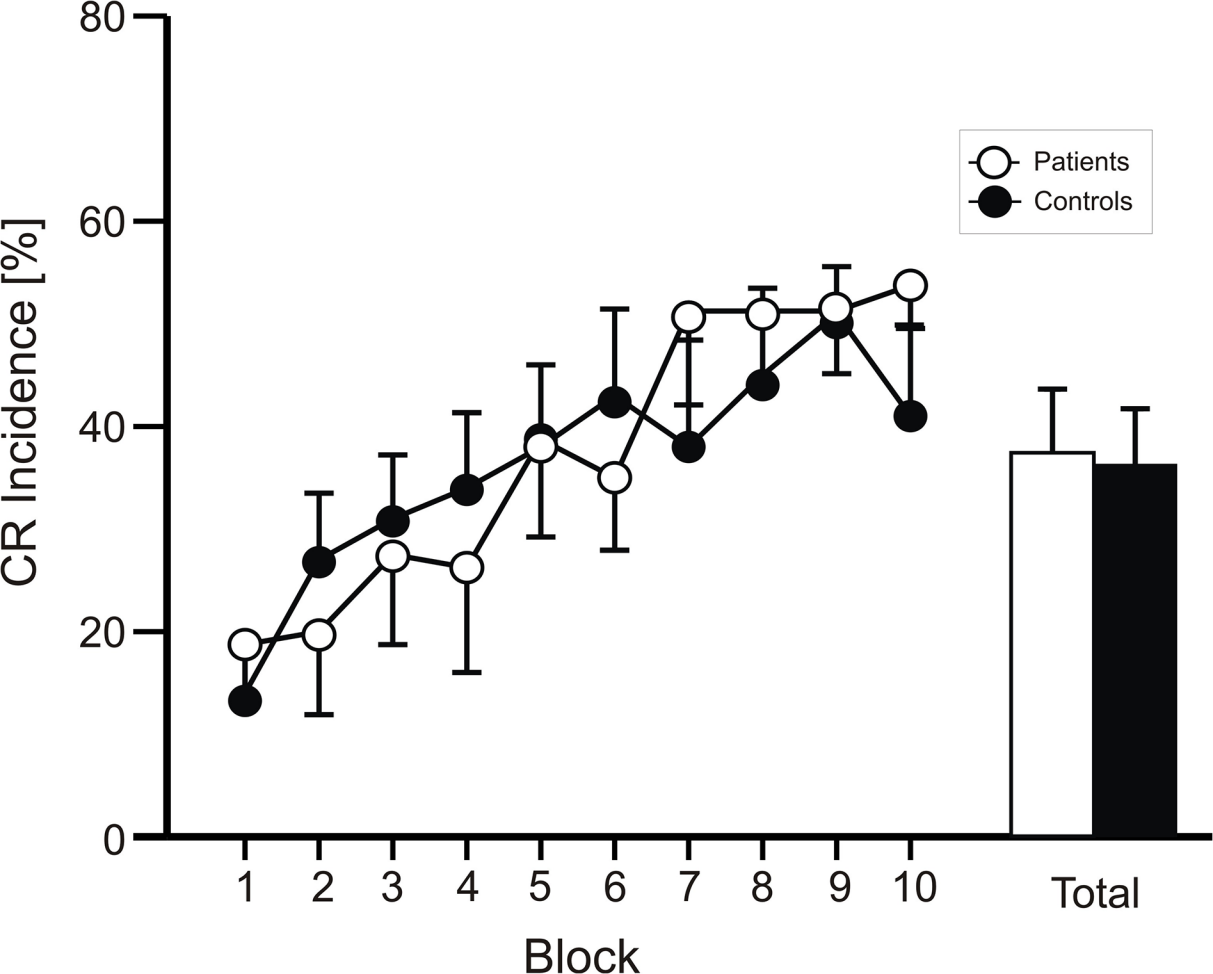
Mean percentage of conditioned responses (CR incidence) in paired (e.g., acquisition) trials. CR incidence and standard errors (SE) are shown per block of ten trials (Total = Mean total percentage CR incidence) in DMD patients (white dots and column) and in control subjects (black dots and column).

Fig 1 shows mean percentage of CR incidences ± standard error (SE) in paired trials in DMD patients (n = 8) and in control subjects (n = 10). Across the ten blocks both groups exhibited a significant increase of percentage CR incidences with a mean total CR incidence of 37.4 ± 17.6% in DMD patients and 36.2 ± 17.3% in controls.

Analysis of variance with percentage of CR incidence as dependent variable, block (1–10) as the within subject factor and group (controls vs. DMD patients) as the between subject factor was calculated. The main effect of group was not significant [F(1,16) = 0.02; p = 0.89]. The block effect was significant [F(9,144) = 9.1; p < 0.001], the block by group interaction effect was not significant [F(9,144) = 1.1; p = 0.38].

**Fig 2:**
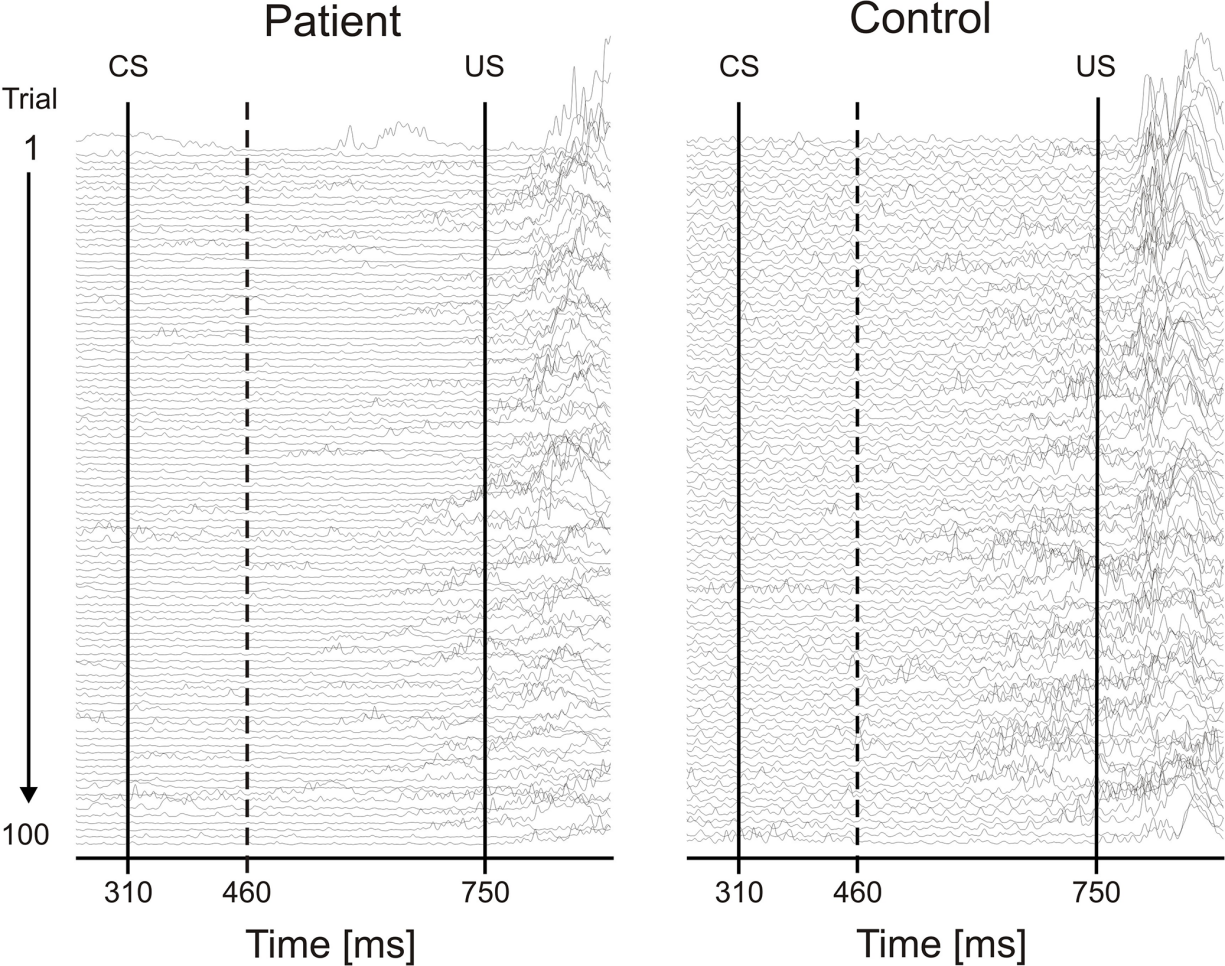
Eyeblink conditioning in an individual DMD patient and control subject. Rectified and filtered EMG data of the orbicularis oculi muscle of 100 paired CS-US trials are shown from the beginning of the experiment (top) to the end (bottom). The first vertical line indicates the CS onset and the second the beginning of the US. Responses occurring within the 150 ms interval after CS onset (dotted line) were considered alpha-responses and were not counted as CRs. See Methods for further details.

Examples of eyeblink recordings in individual subjects are shown to illustrate group findings (Fig 2). EMG data are shown of the 100 paired CS-US trials from the first (top) to the last trial (bottom). CRs are specified by EMG bursts occurring within the relevant CS-US window indicated by the two vertical lines, i.e. beyond the 150 ms interval after CS onset. Examples of a 13 year old DMD patient and a 12 year old control subject are shown. The total percentage CR incidence was 43% and 53%, respectively. CRs started after few paired trials. CRs were exhibited in both the DMD patient as well as the control subject. Individual eyeblink conditioning recordings of all participants are shown as S1, S2, S3 and S4 Figs show findings in DMD patients, S5, S6, S7, S8 and S9 Figs show findings in control subjects.

### CR timing

**Fig 3:**
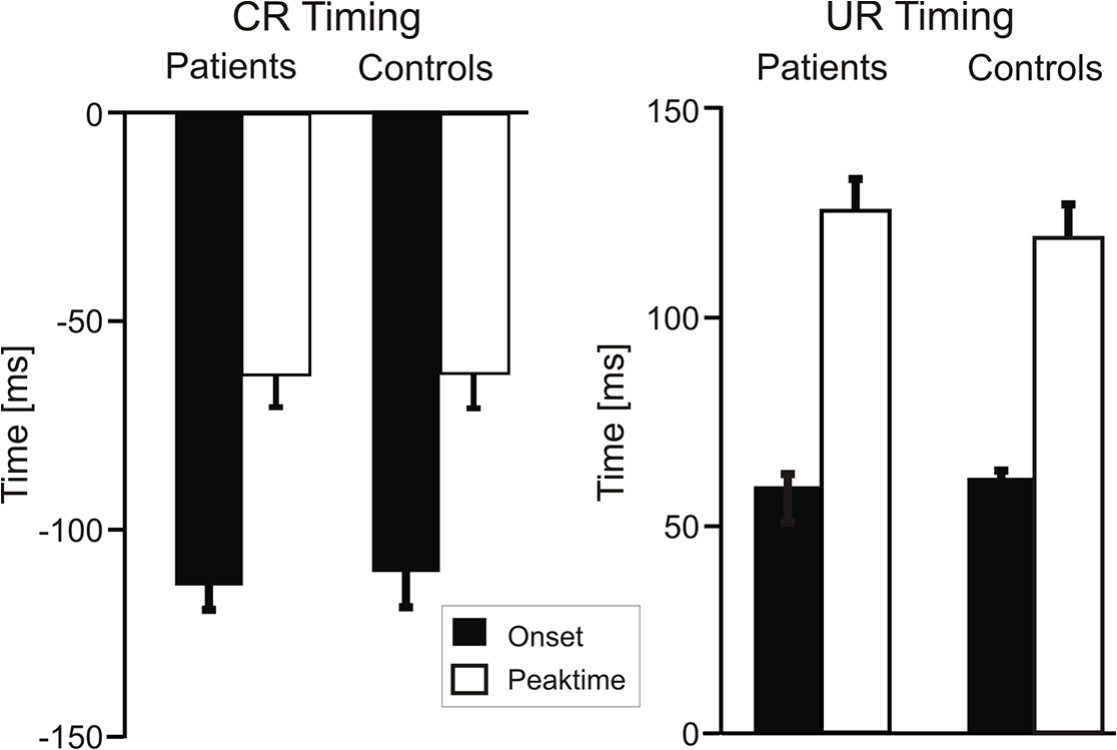
Timing of conditioned and unconditioned eyeblink responses. Mean values and standard deviations (SD) of onset (black columns) and peaktime latencies (white columns) of conditioned eyeblink responses in paired trials and unconditioned eyeblink responses in unpaired trials in DMD patients and control subjects. Values for onset and peaktime refer to the time as related to the onset of the US (air puff), set as 0 ms.

CRs started on average 110 ms prior the onset of the air puff, set as 0 ms (mean CR onset was -113.7 ± 16.5 ms in DMD patients and -110.6 ± 26.6 ms in controls). Mean peaktime latencies were -62.5 ± 21.9 ms in DMD patients and -62.2 ± 28.4 ms in controls (Fig 3). The comparison of timing of conditioned eyeblink responses between DMD patients and controls did not reveal significant differences [CR onset: T(16) = 0.28, p = 0.78; CR peaktime: T(16) = 0.02, p = 0.98; unpaired t test].

### Extinction

In DMD patients there was a decline of CRs comparing the last block of paired trials (block 10) and the extinction block (53.7 ± 11.9% vs. 38.7 ± 15.5%). In control subjects there was no decline comparing block 10 and the extinction block (41.0 ± 28.0% vs. 42.0 ± 16.2%). Controls, however, showed a lower mean CR incidence in block 10 compared to block 9 (41.0 ± 28.0% in block 10 vs. 51.0 ± 14.5% in block 9). ANOVA with percentage CR incidence as dependent variable, block 10 and extinction block as the within subject factor and group as the between subject factor was calculated. The block effect (that is extinction effect) was not significant [F(1,16) = 1.3, p = 0.27]. The group [F(1,16) = 0.47, p = 0.50] and block by group effect were not significant [F(1,16) = 1.7, p = 0.21]. Thus, although both controls and DMD showed a numerical decline during extinction, this did not reach statistical significance.

### Unconditioned eyeblink responses, spontaneous blink-rate and alpha-blinks

Because URs were not recorded in one of the patients for technical reasons, data of 7 patients were analysed. Fig 3 shows mean values of UR onset ± standard deviation (SD) in unpaired trials (59.2 ± 9.3ms in DMD patients; 61.0 ± 6.1ms in controls) and mean UR peaktime latencies (124.9 ± 22.0ms in DMD patients; 118.8 ± 25.1ms in controls). There were no significant group differences [UR onset: T(15) = 0.48, p = 0.63; UR peaktime: T(15) = -0.51, p = 0.61; unpaired t test].

The mean number of spontaneous blinks was recorded at the beginning and at the end of the experiment (DMD patients: at the beginning 10.7 ± 9.6 blinks/min, at the end 18.5 ± 11.2; controls: at the beginning 19.5 ± 10.1 blinks/min, at the end 17.0 ± 10.0). ANOVA with spontaneous blink rate as depending variable, at the beginning and at the end of the experiment as within subject factor and group as between subject factor did not reveal a significant time (before vs. after conditioning) [F(1,16) = 1.0; p = 0.33], time by group interaction effect, [F(1,16) = 3.8; p = 0.068] or group effect [F(1,16) = 0.79; p = 0.39]. The rate of alpha-blinks did not differ between groups [T(15) = 0.06, p = 0.95; unpaired t test].

## Discussion

In the present study delay eyeblink conditioning was used to assess cerebellar function in DMD patients. The acquisition of conditioned eyeblink responses was not significantly reduced as compared to healthy controls. In addition timing of conditioned responses was not disturbed. At first glance, findings appear to contradict studies pointing to disordered cerebellar function in DMD.

In agreement with animal data disturbed acquisition and timing of CRs has been shown in patients with cerebellar disorders [40, 42, 43, 45–47]. Moreover, reduced CR acquisition has been reported in disorders with subtle and even subclinical cerebellar signs [34–38]. Because of the marked loss of dystrophin in the cerebellar cortex revealed by animal studies and its detrimental effect on plasticity in the cerebellar cortex underlying learning [5–9], impaired eyeblink conditioning was expected in DMD as well. This, however, was not the case.

DMD may affect cerebellar areas which are involved in cognitive processes but not the cerebellar areas which are critical in eyeblink conditioning. Animal and human lesion studies show a functional compartmentalization within the cerebellum [48]. For example the medial cerebellum contributes to posture, gait and oculomotor control whereas the intermediate cerebellum is important for limb coordination. Eyeblink conditioning has also been shown to depend on the integrity of the intermediate cerebellum [49]. Cognitive functions on the other hand are thought to be supported by the newer parts of the cerebellum that is the posterolateral hemispheres [17, 18].

In fact, recent findings in dystrophin deficient mdx mice suggest that these cerebellar regions are differently affected by the Duchenne pathology. Using immunohistochemistry it has been shown that the density of dystrophin is higher in the somatic and dendritic membranes of Purkinje cells in the lateral parts of the cerebellar hemisphere than in the vermis [50]. Moreover, altered intrinsic membrane properties were reported. Whereas control mice showed enhanced firing rates in the lateral cerebellum compared to vermal regions, this regional difference was abolished in mdx mice by significantly reduced action potential activity and firing frequency of Purkinje cells from the lateral cerebellum [51]. Findings support the view that the predominant loss of dystrophin within the lateral cerebellum may contribute to cognitive dysfunction in DMD by specifically alteration of cerebro-cerebellar loops. The present findings of preserved eyeblink conditioning suggest that the intermediate cerebellum is largely preserved in DMD, and further support findings that the lateral cerebellum is primarily affected. However, we did not perform neuropsychological testing and the present patients were not characterised from a cognitive point of view. As a further limitation, the number of DMD patients was small. Therefore, it cannot be excluded that disordered eyeblink conditioning may be present in a larger group of patients with proven cognitive deficits. However, findings of the animal studies discussed above, which show prominent involvement of the lateral cerebellum in DMD, do not support this assumption.

In addition to unaffected CR acquisition timing of conditioned responses was not disturbed in the present DMD patients. A shortened onset of responses has been reported following lesions of the anterior cerebellar lobe in animals and humans [41, 52]. However, there are other conditions showing reduced CR acquisition which were not accompanied by disordered CR timing, e.g. in patients with essential tremor and in migraine [34, 38]. Furthermore, extinction was not different from controls. Note that although both controls and patients showed a numerical decline of conditioned responses during extinction, this was not significant. The reason may be young age and the relatively small number of extinction trials. Extinction trials were restricted to ten because most of the patients were too disabled to perform a longer session. Age-related and developmental changes of the acquisition of eyeblink conditioning are well known [53–55]. This may equally apply to extinction which is known to be, at least in part, an active process of unlearning [56]. As yet, only few animal studies have assessed age differences in extinction. Different to our findings, rats at a younger age showed more rapid extinction than older rats. Extinction, however, was tested 24 hours after acquisition, and differences in extinction were related to impaired retention at a younger age [57]. The present findings need to be confirmed in future studies in larger groups of children of different ages and using more extinction trials.

In conclusion the present study does not reveal evidence for impaired eyeblink conditioning in DMD subjects. The intermediate cerebellum may be spared in DMD. Findings, however, do not exclude a possible role of the lateral cerebellum in cognitive dysfunction in DMD, which may be primarily affected.

## Supporting Information

S1 Fig Eyeblink conditioning in DMD patients 1 and 2. Rectified and filtered EMG data of the orbicularis oculi muscle of 100 paired CS-US trials are shown from the beginning of the experiment (top) to the end (bottom). The first vertical line indicates the CS onset and the second the beginning of the US. Responses occurring within the 150 ms interval after CS onset (dotted line) were considered alpha-responses and were not counted as CRs. The patient numbers correspond to the numbers in Table 1.
(JPG)

S2 Fig Eyeblink conditioning in DMD patients 3 and 4. Rectified and filtered EMG data of the orbicularis oculi muscle of 100 paired CS-US trials are shown from the beginning of the experiment (top) to the end (bottom). The first vertical line indicates the CS onset and the second the beginning of the US. Responses occurring within the 150 ms interval after CS onset (dotted line) were considered alpha-responses and were not counted as CRs. The patient numbers correspond to the numbers in Table 1.
(JPG)

S3 Fig Eyeblink conditioning in DMD patients 5 and 6. Rectified and filtered EMG data of the orbicularis oculi muscle of 100 paired CS-US trials are shown from the beginning of the experiment (top) to the end (bottom). The first vertical line indicates the CS onset and the second the beginning of the US. Responses occurring within the 150 ms interval after CS onset (dotted line) were considered alpha-responses and were not counted as CRs. The patient numbers correspond to the numbers in Table 1.
(JPG)

S4 Fig Eyeblink conditioning in DMD patients 7 and 8. Rectified and filtered EMG data of the orbicularis oculi muscle of 100 paired CS-US trials are shown from the beginning of the experiment (top) to the end (bottom). The first vertical line indicates the CS onset and the second the beginning of the US. Responses occurring within the 150 ms interval after CS onset (dotted line) were considered alpha-responses and were not counted as CRs. The patient numbers correspond to the numbers in Table 1.
(JPG)

S5 Fig Eyeblink conditioning in control subjects 1 and 2. Rectified and filtered EMG data of the orbicularis oculi muscle of 100 paired CS-US trials are shown from the beginning of the experiment (top) to the end (bottom). The first vertical line indicates the CS onset and the second the beginning of the US. Responses occurring within the 150 ms interval after CS onset (dotted line) were considered alpha-responses and were not counted as CRs.
(JPG)

S6 Fig Eyeblink conditioning in control subjects 3 and 4. Rectified and filtered EMG data of the orbicularis oculi muscle of 100 paired CS-US trials are shown from the beginning of the experiment (top) to the end (bottom). The first vertical line indicates the CS onset and the second the beginning of the US. Responses occurring within the 150 ms interval after CS onset (dotted line) were considered alpha-responses and were not counted as CRs.
(JPG)

S7 Fig Eyeblink conditioning in control subjects 5 and 6. Rectified and filtered EMG data of the orbicularis oculi muscle of 100 paired CS-US trials are shown from the beginning of the experiment (top) to the end (bottom). The first vertical line indicates the CS onset and the second the beginning of the US. Responses occurring within the 150 ms interval after CS onset (dotted line) were considered alpha-responses and were not counted as CRs.
(JPG)

S8 Fig Eyeblink conditioning in control subjects 7 and 8. Rectified and filtered EMG data of the orbicularis oculi muscle of 100 paired CS-US trials are shown from the beginning of the experiment (top) to the end (bottom). The first vertical line indicates the CS onset and the second the beginning of the US. Responses occurring within the 150 ms interval after CS onset (dotted line) were considered alpha-responses and were not counted as CRs.
(JPG)

S9 Fig Eyeblink conditioning in control subjects 9 and 10. Rectified and filtered EMG data of the orbicularis oculi muscle of 100 paired CS-US trials are shown from the beginning of the experiment (top) to the end (bottom). The first vertical line indicates the CS onset and the second the beginning of the US. Responses occurring within the 150 ms interval after CS onset (dotted line) were considered alpha-responses and were not counted as CRs.
(JPG)
