# Supplementary figures and images for: Cerebellar-Dependent Associative Learning Is Preserved in Duchenne Muscular Dystrophy: A Study Using Delay Eyeblink Conditioning
Source: PLoS One. 2015 May 14;10(5):e0126528. doi: 10.1371/journal.pone.0126528 (PMC4431835; doi:10.1371/journal.pone.0126528)

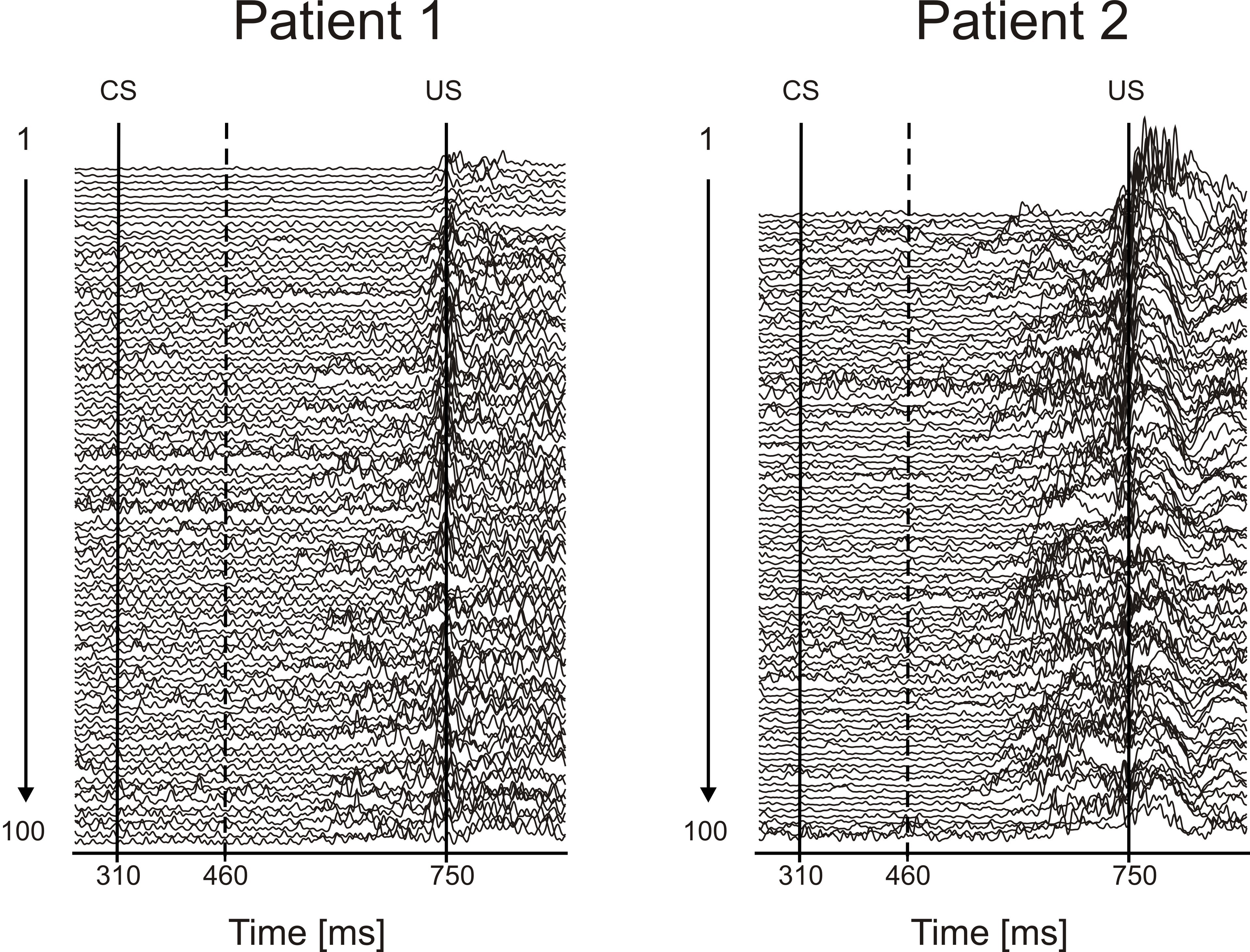

Supplement: S1 Fig — Rectified and filtered EMG data of the orbicularis oculi muscle of 100 paired CS-US trials are shown from the beginning of the experiment (top) to the end (bottom). The first vertical line indicates the CS onset and the second the beginning of the US. Responses occurring within the 150 ms interval after CS onset (dotted line) were considered alpha-responses and were not counted as CRs. The patient numbers correspond to the numbers in Table 1. (JPG) [file pone.0126528.s001.jpg]

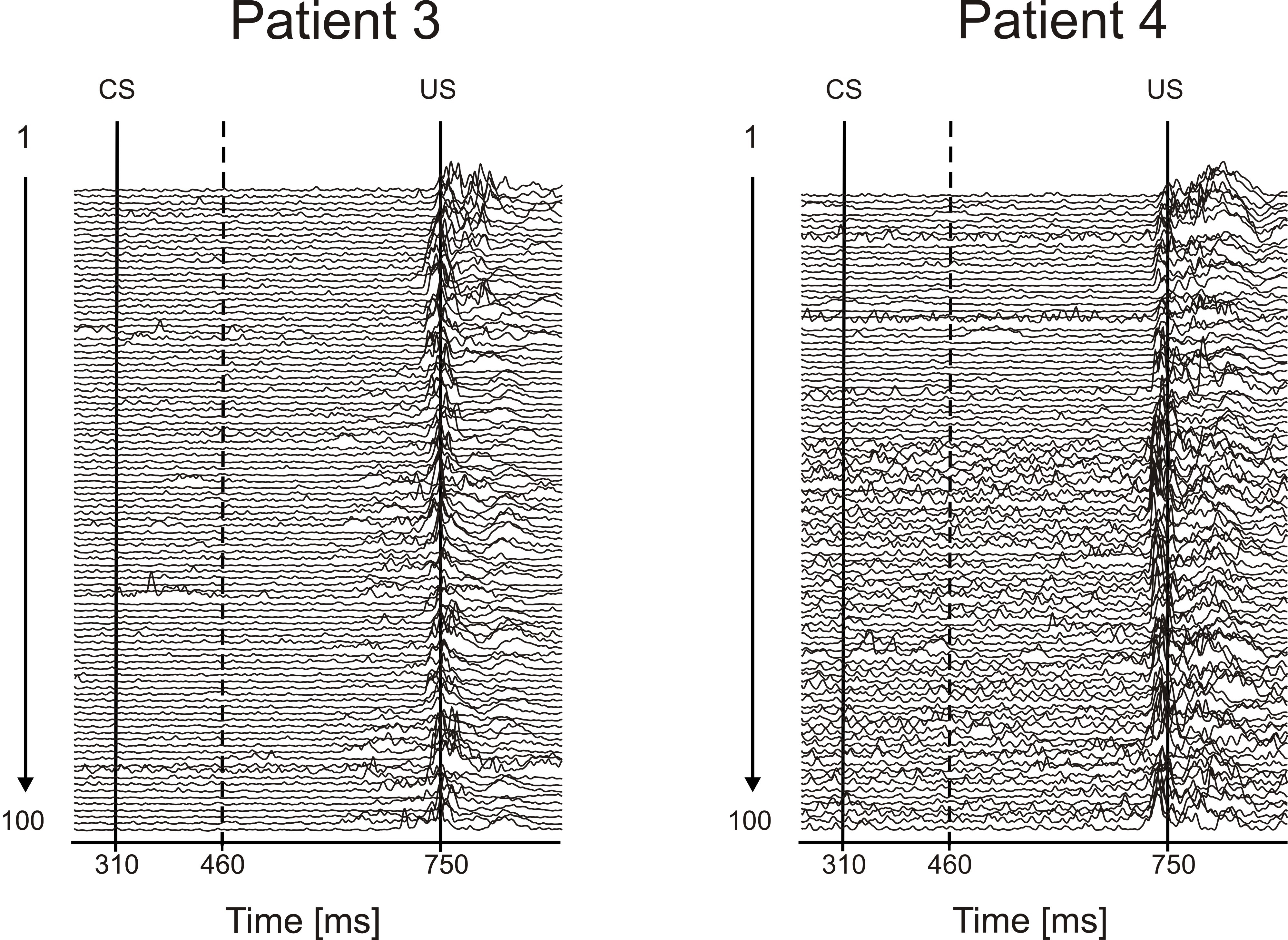

Supplement: S2 Fig — Rectified and filtered EMG data of the orbicularis oculi muscle of 100 paired CS-US trials are shown from the beginning of the experiment (top) to the end (bottom). The first vertical line indicates the CS onset and the second the beginning of the US. Responses occurring within the 150 ms interval after CS onset (dotted line) were considered alpha-responses and were not counted as CRs. The patient numbers correspond to the numbers in Table 1. (JPG) [file pone.0126528.s002.jpg]

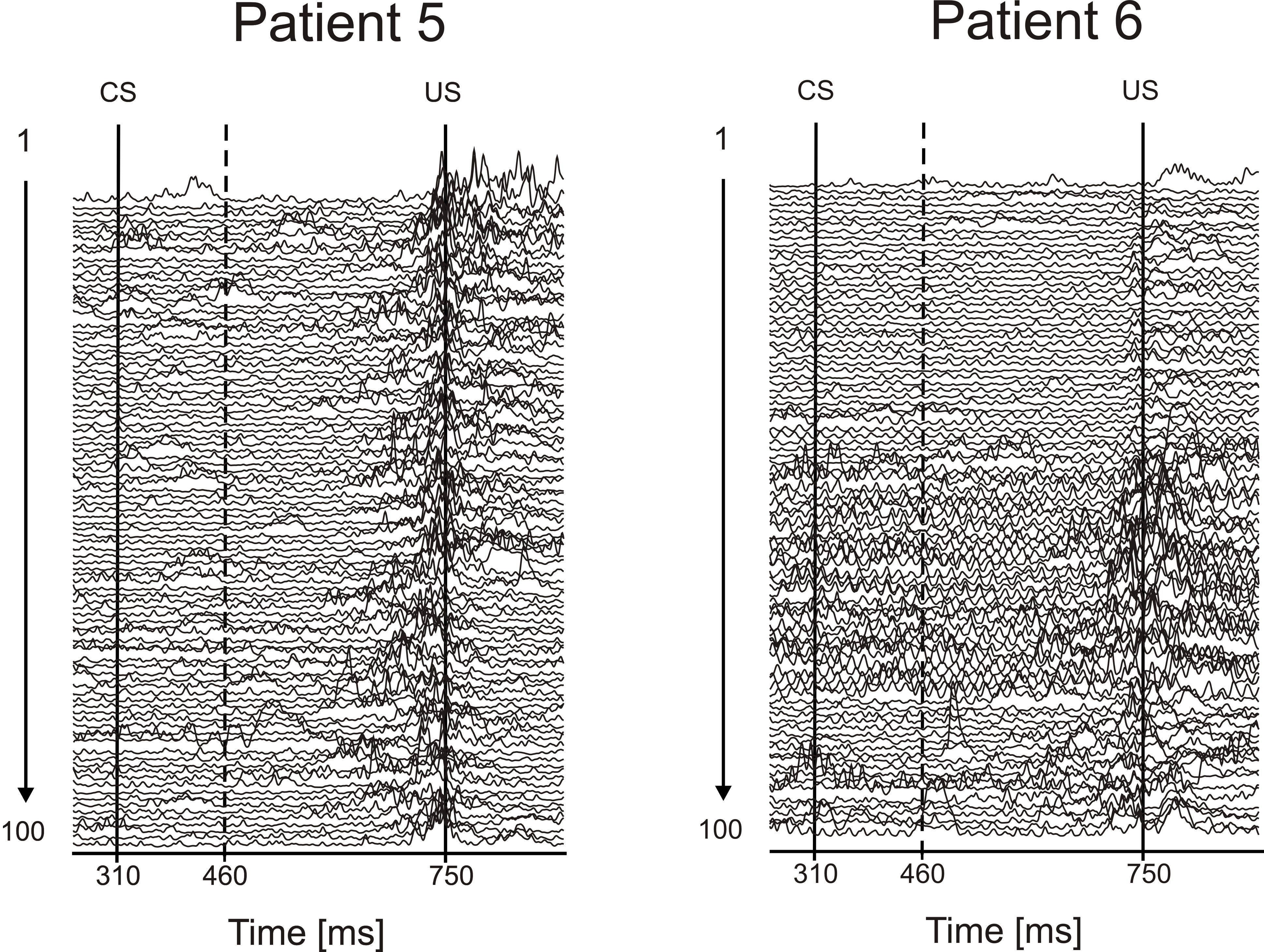

Supplement: S3 Fig — Rectified and filtered EMG data of the orbicularis oculi muscle of 100 paired CS-US trials are shown from the beginning of the experiment (top) to the end (bottom). The first vertical line indicates the CS onset and the second the beginning of the US. Responses occurring within the 150 ms interval after CS onset (dotted line) were considered alpha-responses and were not counted as CRs. The patient numbers correspond to the numbers in Table 1. (JPG) [file pone.0126528.s003.jpg]

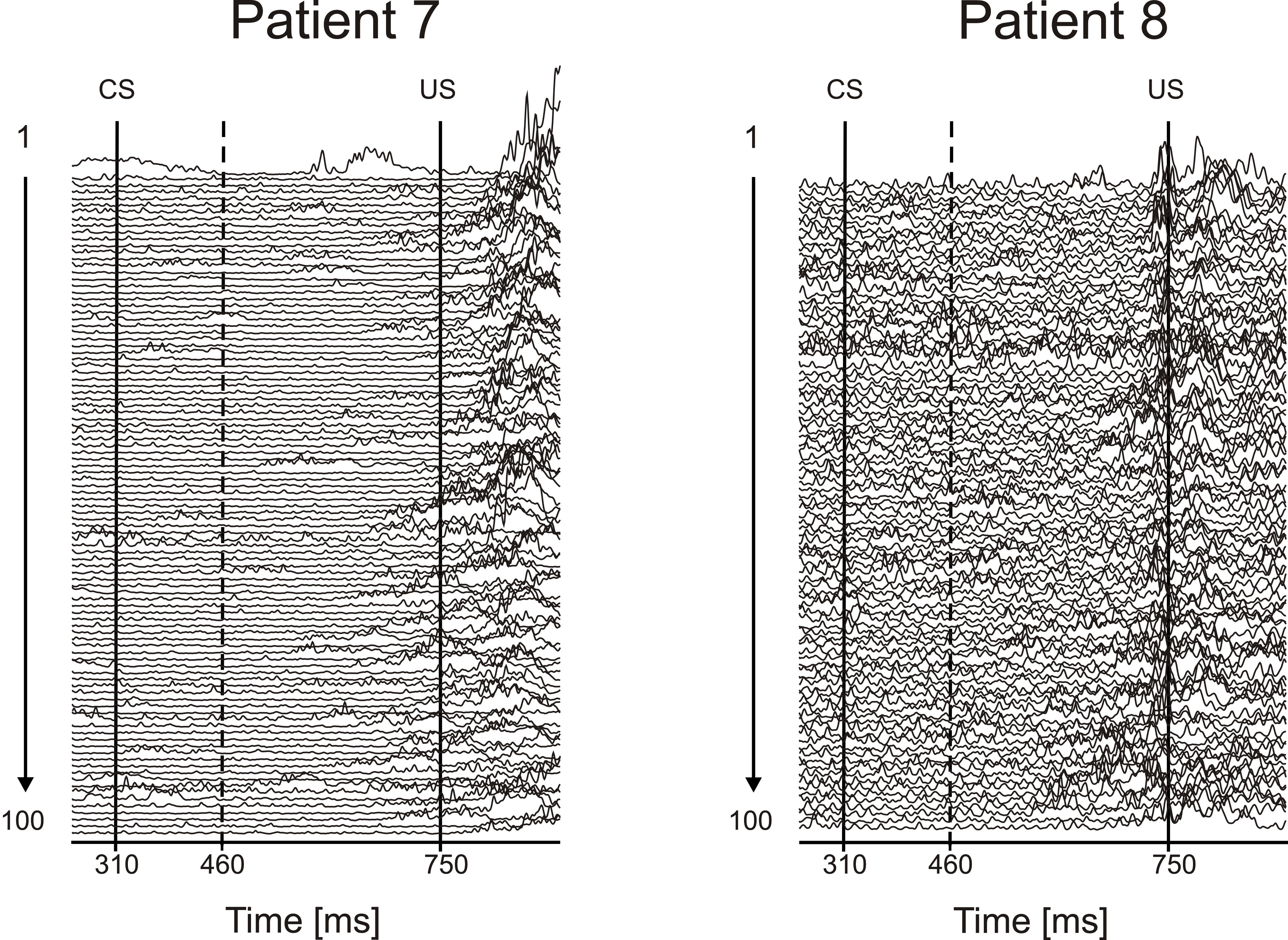

Supplement: S4 Fig — Rectified and filtered EMG data of the orbicularis oculi muscle of 100 paired CS-US trials are shown from the beginning of the experiment (top) to the end (bottom). The first vertical line indicates the CS onset and the second the beginning of the US. Responses occurring within the 150 ms interval after CS onset (dotted line) were considered alpha-responses and were not counted as CRs. The patient numbers correspond to the numbers in Table 1. (JPG) [file pone.0126528.s004.jpg]

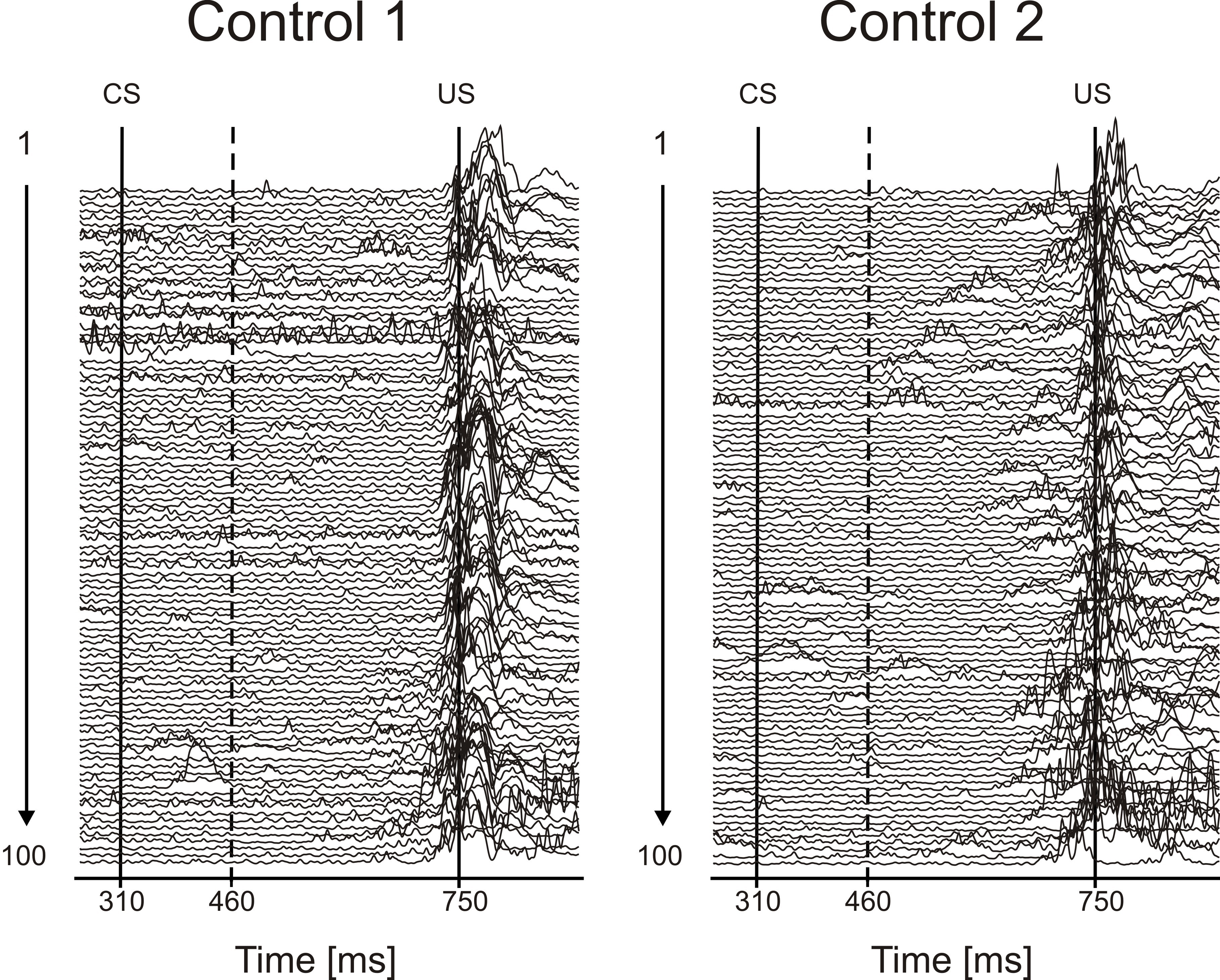

Supplement: S5 Fig — Rectified and filtered EMG data of the orbicularis oculi muscle of 100 paired CS-US trials are shown from the beginning of the experiment (top) to the end (bottom). The first vertical line indicates the CS onset and the second the beginning of the US. Responses occurring within the 150 ms interval after CS onset (dotted line) were considered alpha-responses and were not counted as CRs. (JPG) [file pone.0126528.s005.jpg]

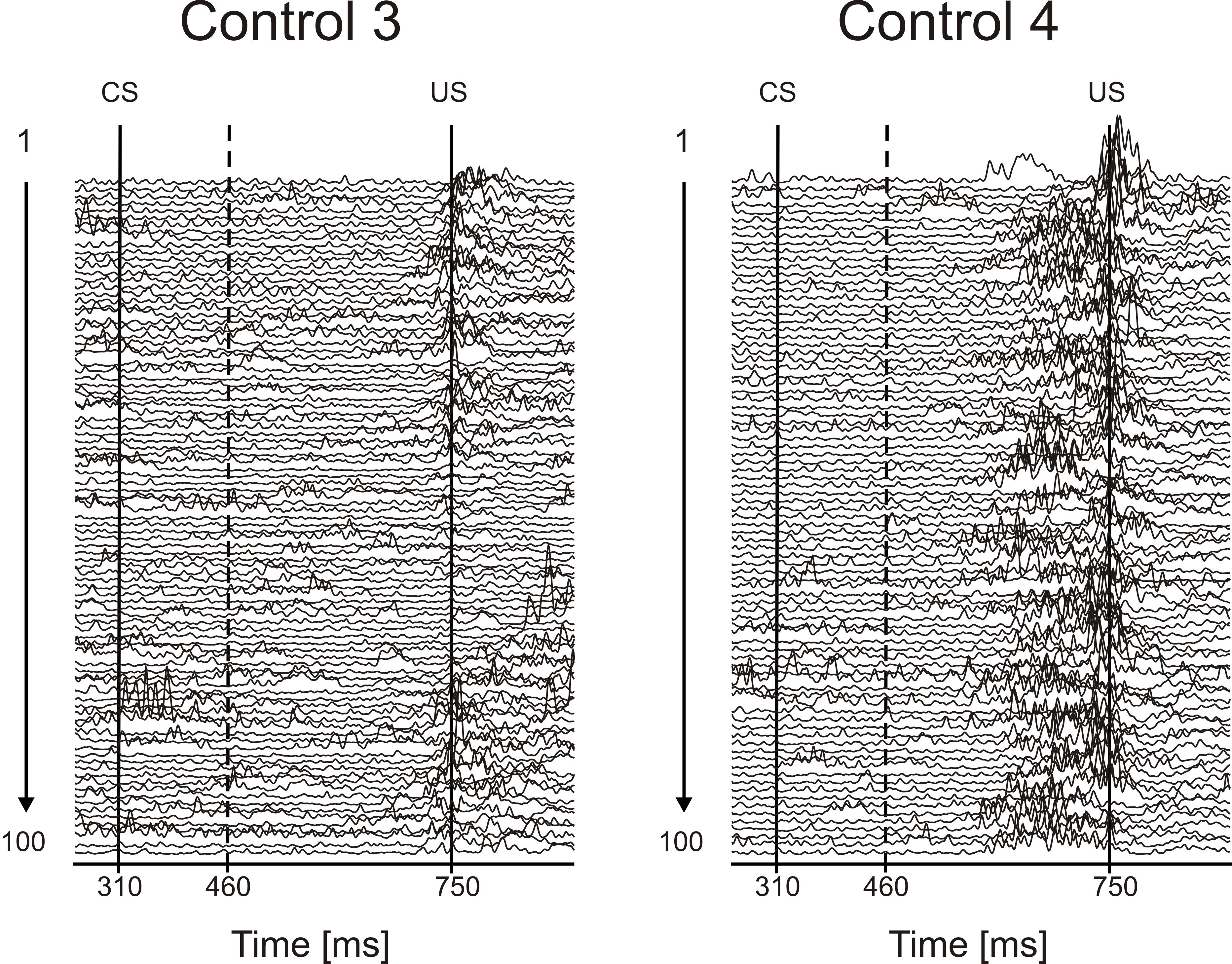

Supplement: S6 Fig — Rectified and filtered EMG data of the orbicularis oculi muscle of 100 paired CS-US trials are shown from the beginning of the experiment (top) to the end (bottom). The first vertical line indicates the CS onset and the second the beginning of the US. Responses occurring within the 150 ms interval after CS onset (dotted line) were considered alpha-responses and were not counted as CRs. (JPG) [file pone.0126528.s006.jpg]

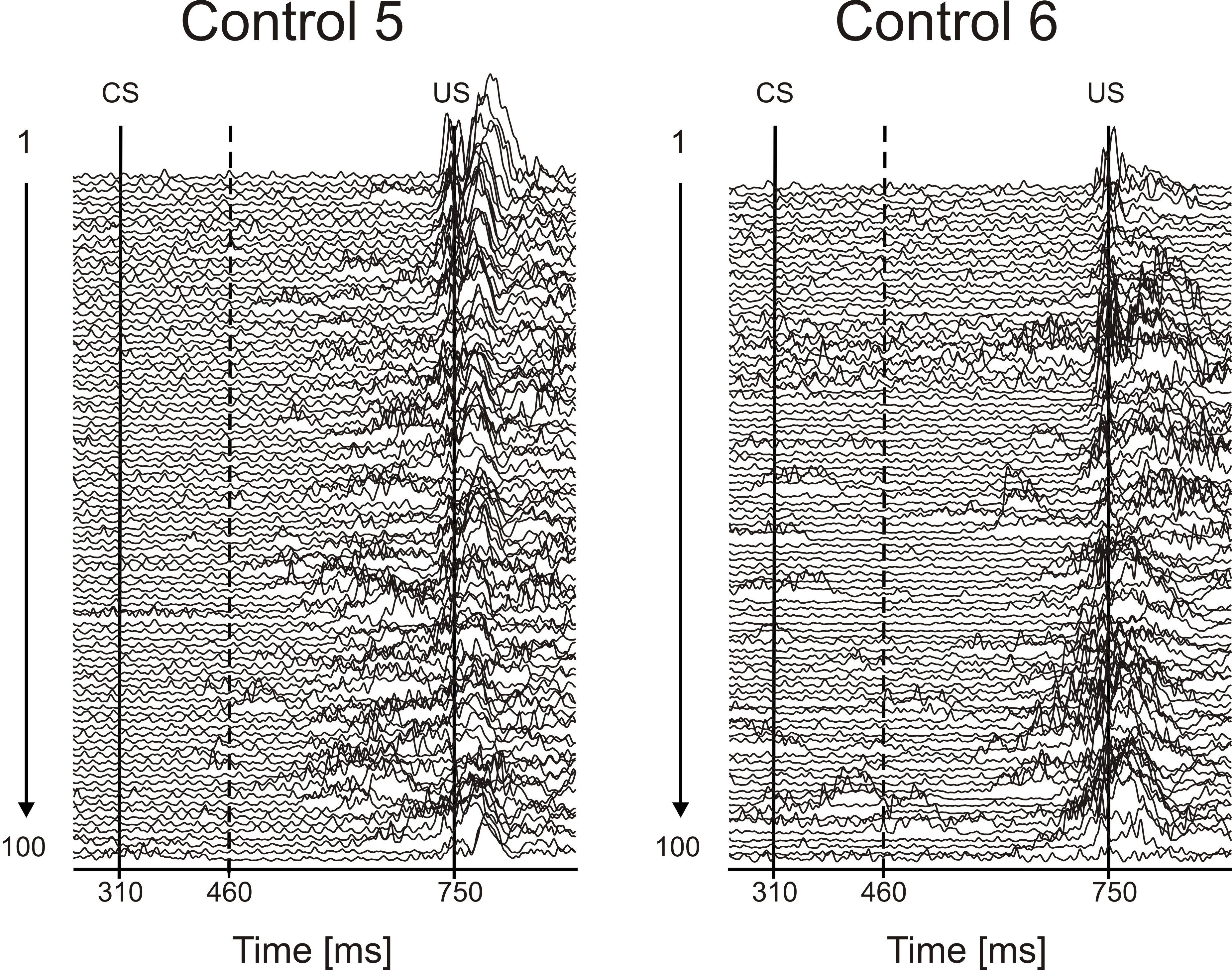

Supplement: S7 Fig — Rectified and filtered EMG data of the orbicularis oculi muscle of 100 paired CS-US trials are shown from the beginning of the experiment (top) to the end (bottom). The first vertical line indicates the CS onset and the second the beginning of the US. Responses occurring within the 150 ms interval after CS onset (dotted line) were considered alpha-responses and were not counted as CRs. (JPG) [file pone.0126528.s007.jpg]

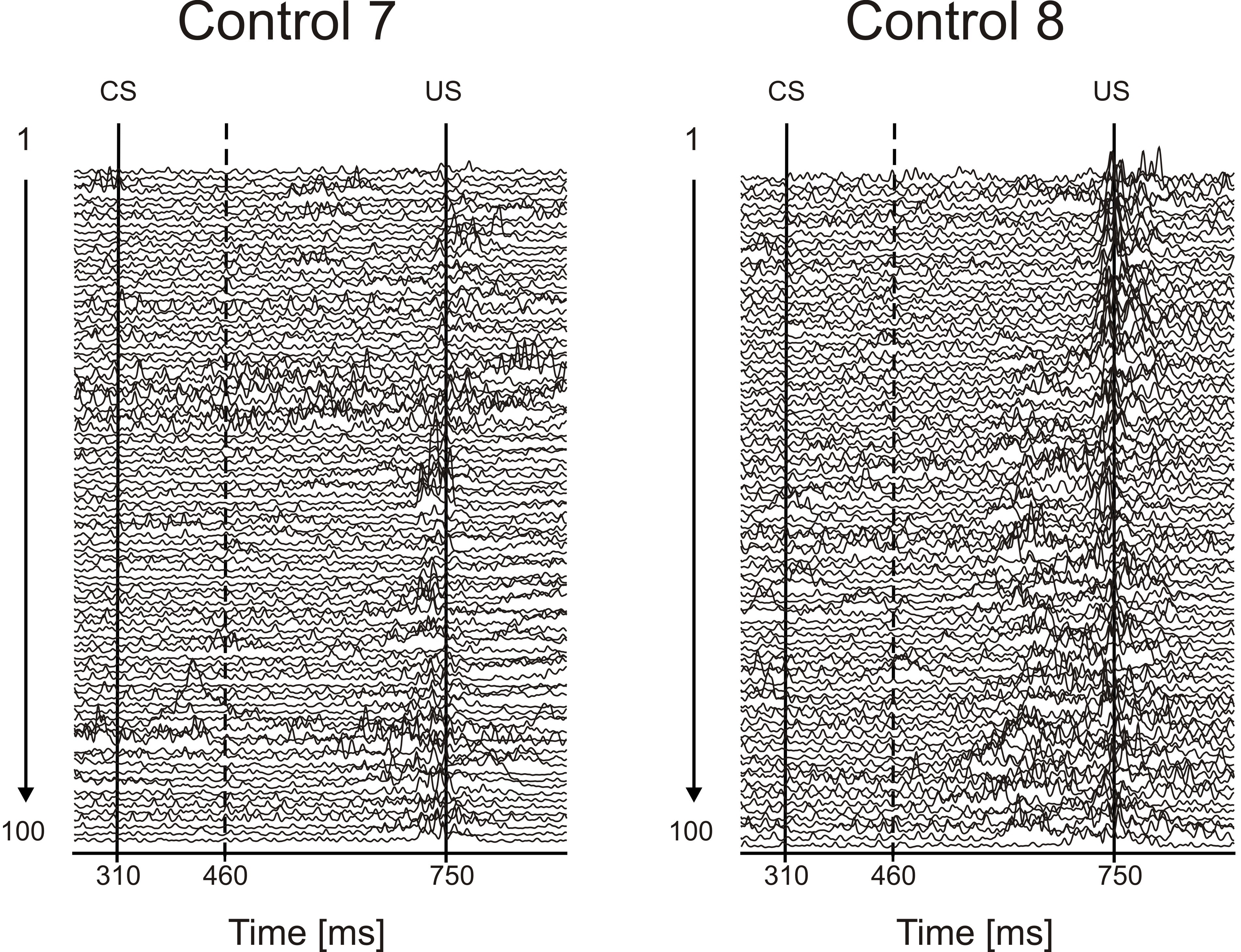

Supplement: S8 Fig — Rectified and filtered EMG data of the orbicularis oculi muscle of 100 paired CS-US trials are shown from the beginning of the experiment (top) to the end (bottom). The first vertical line indicates the CS onset and the second the beginning of the US. Responses occurring within the 150 ms interval after CS onset (dotted line) were considered alpha-responses and were not counted as CRs. (JPG) [file pone.0126528.s008.jpg]

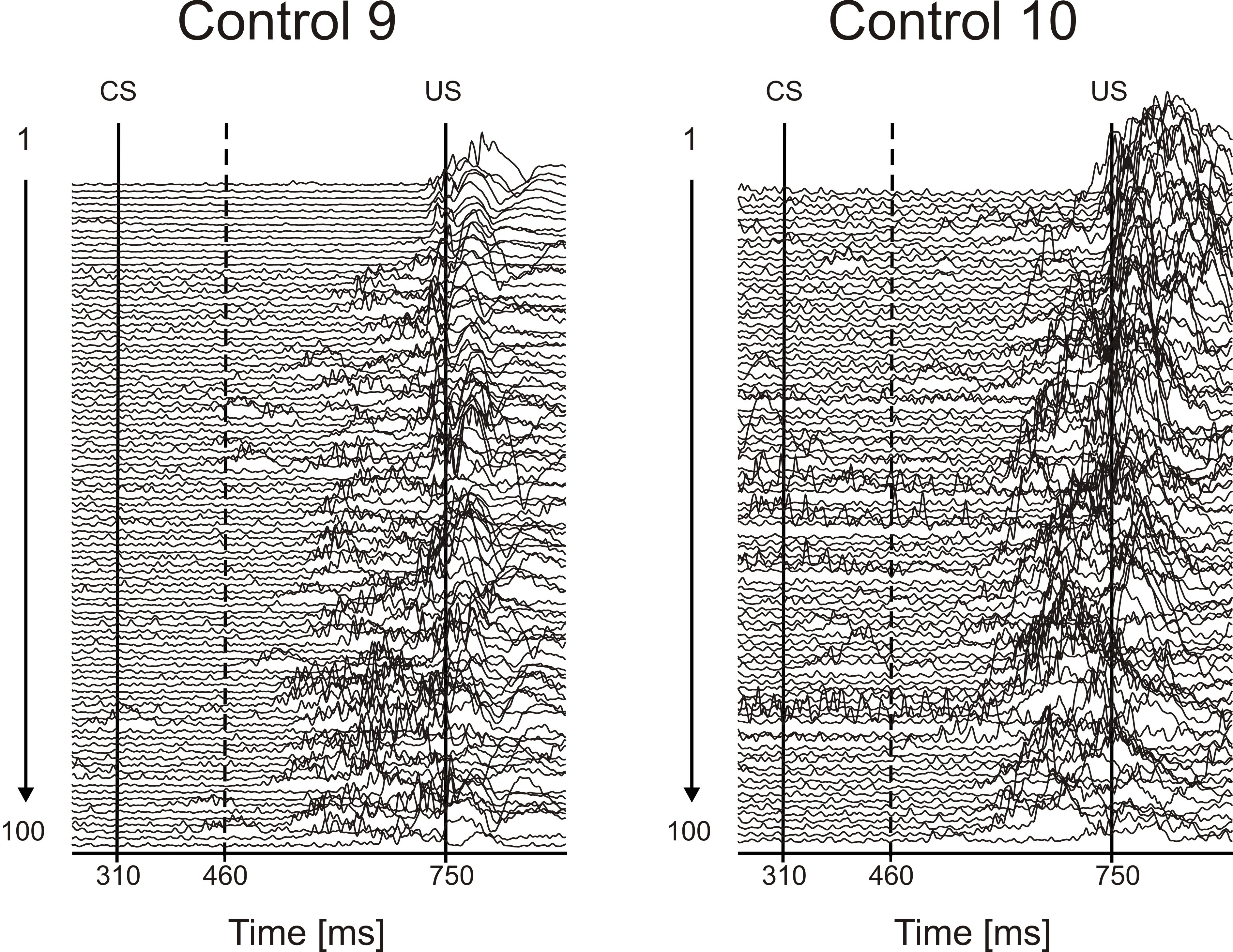

Supplement: S9 Fig — Rectified and filtered EMG data of the orbicularis oculi muscle of 100 paired CS-US trials are shown from the beginning of the experiment (top) to the end (bottom). The first vertical line indicates the CS onset and the second the beginning of the US. Responses occurring within the 150 ms interval after CS onset (dotted line) were considered alpha-responses and were not counted as CRs. (JPG) [file pone.0126528.s009.jpg]
